# Association of Food Allergy and Other Allergic Conditions With Autism Spectrum Disorder in Children

**DOI:** 10.1001/jamanetworkopen.2018.0279

**Published:** 2018-06-08

**Authors:** Guifeng Xu, Linda G. Snetselaar, Jin Jing, Buyun Liu, Lane Strathearn, Wei Bao

**Affiliations:** 1 Department of Epidemiology, College of Public Health, University of Iowa, Iowa City; 2 Center for Disabilities and Development, University of Iowa Stead Family Children’s Hospital, Iowa City; 3 Department of Maternal and Child Health, School of Public Health, Sun Yat-Sen University, Guangzhou, China; 4 Division of Developmental and Behavioral Pediatrics, Stead Family Department of Pediatrics, University of Iowa Carver College of Medicine, Iowa City

## Abstract

**Importance:**

The prevalence of autism spectrum disorder (ASD) in US children has increased during the past decades. Immunologic dysfunction has recently emerged as a factor associated with ASD. Although children with ASD are more likely to have gastrointestinal disorders, little is known about the association between food allergy and ASD.

**Objective:**

To examine the association of food allergy and other allergic conditions with ASD in US children.

**Design, Setting, and Participants:**

This population-based, cross-sectional study used data from the National Health Interview Survey collected between 1997 and 2016. The data analysis was performed in 2018. All eligible children aged 3 to 17 years were included. Food allergy, respiratory allergy, and skin allergy were defined based on an affirmative response in the questionnaire by a parent or guardian.

**Main Outcomes and Measures:**

Reported ASD diagnosed by a physician or other health professional.

**Results:**

This analysis included 199 520 children (unweighted mean [SD] age, 10.21 [4.41] years; 102 690 boys [51.47%]; 55 476 Hispanic [27.80%], 97 200 non-Hispanic white [48.72%], 30 760 non-Hispanic black [15.42%], and 16 084 non-Hispanic other race [8.06%]). Among them, 8734 (weighted prevalence, 4.31%) had food allergy, 24 555 (12.15%) had respiratory allergy, and 19 399 (9.91%) had skin allergy. A diagnosis of ASD was reported in 1868 children (0.95%). The weighted prevalence of reported food, respiratory, and skin allergies was higher in children with ASD (11.25%, 18.73%, and 16.81%, respectively) compared with children without ASD (4.25%, 12.08%, and 9.84%, respectively). In analyses adjusting for age, sex, race/ethnicity, family highest education level, family income level, geographical region, and mutual adjustment for other allergic conditions, the associations between allergic conditions and ASD remained significant. The odds ratio (OR) of ASD increased in association with food allergy (OR, 2.29; 95% CI, 1.87-2.81), respiratory allergy (OR, 1.28; 95% CI, 1.10-1.50), and skin allergy (OR, 1.50; 95% CI, 1.28-1.77) when comparing children with these conditions and those without.

**Conclusions and Relevance:**

In a nationally representative sample of US children, a significant and positive association of common allergic conditions, in particular food allergy, with ASD was found. Further investigation is warranted to elucidate the causality and underlying mechanisms.

## Key Points

**Question:**

What are the associations of food allergy and other allergic conditions with autism spectrum disorder (ASD) in children?

**Findings:**

This cross-sectional study used nationally representative data from 199 520 children aged 3 to 17 years who participated in the US National Health Interview Survey from 1997 to 2016. Children with food, respiratory, and skin allergies were significantly more likely to have ASD than children without these allergies.

**Meaning:**

Common allergic conditions, in particular food allergy, are associated with ASD among US children, but the underlying mechanism for this association needs further study.

## Teaser

This cross-sectional study uses data from the National Health Interview Survey to examine the association of food, respiratory, and skin allergies with autism spectrum disorder (ASD) in US children.

## Introduction

Autism spectrum disorder (ASD) is a complex neurodevelopmental disorder characterized by deficits in social interaction, language, and communication, as well as the presence of restricted repetitive behaviors. The prevalence of ASD in US children has steadily increased over the past decades.^1,2,3,4^ The Autism and Developmental Disabilities Monitoring Network reported that the estimated prevalence of ASD increased from 0.67% in 2000 to 1.46% in 2012.^1^ A recent study using data from the National Health Interview Survey (NHIS) found that the prevalence of ASD in US children aged 3 to 17 years was 2.24% in 2014, 2.41% in 2015, and 2.76% in 2016.^3^ A similar upward trend in ASD prevalence has been observed in many other developed and developing countries worldwide.^5^ The etiology of ASD involves both genetic and environmental risk factors,^5,6^ and it is estimated that up to 40% to 50% of variance in ASD liability might be attributed to environmental risk factors.^7^

Immunologic dysfunction is a potential link between environmental risk factors and ASD.^8,9,10^ Symptoms of immune function abnormalities, such as frequent infections and increased prevalence of autoimmune conditions, were frequently reported among children with ASD.^5,6,11^ Moreover, maternal infection, inflammatory cytokines, and autoimmune diseases during pregnancy were also associated with ASD in children in some studies.^5^ Available evidence has shown that early-life immune activation can adversely influence certain neurodevelopmental processes, such as neuron growth, through multiple pathways, including altered expression of cytokines and chemokines.^12^

Allergic conditions, including respiratory allergy, skin allergy, and food allergy, are common medical conditions of immunologic dysfunction in children.^13^ Among US children, while there was no significant change in respiratory allergy prevalence from 1997 to 2011, the prevalence of food and skin allergies has increased steadily.^13^ A few previous studies have examined the association between allergic conditions and ASD. However, their results have been inconsistent and inconclusive, and most of those studies focused on respiratory allergy and skin allergy.^14,15^

Animal studies have reported that food allergy in mice can induce autistic-like behavioral changes, including reduced social interaction, increased repetitive behavior, and impaired spatial memory.^16,17^ Although gastrointestinal disorders are more common in children with ASD than those with typical development,^18^ epidemiologic evidence for the association between food allergy and ASD in humans is sparse.^11^ Limited and suggestive evidence indicates that food allergy is related to increased irritability and poorer functional outcomes in ASD^19^ and that exclusion of hyperallergenic foods might improve behavior in children with ASD.^20,21^ Therefore, in this study, we analyzed nationally representative data to examine the associations of food allergy and other allergic conditions with ASD in US children. We hypothesized that children with food allergy may have a higher risk for ASD than children without food allergy.

## Methods

This study was conducted and reported in accordance with the Strengthening the Reporting of Observational Studies in Epidemiology (STROBE) reporting guideline.^22^

### Study Population

The NHIS is a continuous, ongoing, nationally representative annual health survey in the United States.^23,24^ It is conducted by the National Center for Health Statistics (NCHS) at the Centers for Disease Control and Prevention. Since 1957, the NHIS has become the principal source of information on health conditions in the US population.^25^ The NHIS is a survey of a nationally representative sample of the US civilian noninstitutionalized population, with survey information collected via an in-person household interview. It uses a multistage probability sample design. A detailed description of the survey design, methods, and sample weights in the NHIS was published elsewhere.^23,24^ Annual sample size of the NHIS is about 35 000 households containing about 87 500 persons. In the NHIS from 1997 to 2016, the total household response rate ranged from 67.9% to 91.8%, and the conditional response rate for the sample child component ranged from 85.6% to 93.3%. The NHIS was approved by the Research Ethics Review Board of the NCHS and the US Office of Management and Budget. All respondents provided oral informed consent prior to participation. Data from NHIS are released annually and are publicly available online. The University of Iowa institutional review board determined that the current study was exempt based on the use of deidentified data.

### Ascertainment of Variables

The NHIS collected data on a broad range of health topics for all household members, including children. For each interviewed family in the household, 1 sample child (if any children aged ≤17 years were present) was randomly selected by the field representative’s computer.^24^ Detailed health-related information, including information on physical and mental health, was collected for the sample child. This information was provided by an adult, usually a parent, who was knowledgeable about the child’s health.

We included all the children aged 3 to 17 years in the NHIS whose information about allergic conditions and ASD was available. Allergic conditions were defined based on an affirmative response to the following questions^13^: during the past 12 months, has your child had (1) any kind of food or digestive allergy; (2) any kind of respiratory allergy; (3) eczema or any kind of skin allergy? Autism spectrum disorder was defined based on an affirmative response to a question asking whether the sample child received a diagnosis of ASD from a physician or other health professional.^2,3^ From 1997 to 2013, this question was asked as part of a 10-condition checklist. In 2014 onward, the question became a stand-alone item and the wording was revised to name specific conditions, including autism, Asperger disorder, pervasive developmental disorder, and ASD.^2^ Over the 20-year period from 1997 to 2016, 99.82% of participants responded to the question about food allergy; 99.71%, respiratory allergy; 99.86%, skin allergy; and 99.94%, ASD.

Demographic data, including age, sex, race/ethnicity, education, family income, and geographic region, were collected using a standardized questionnaire during the interview. Race and Hispanic ethnicity were self-reported and classified based on the 1997 Office of Management and Budget Standards. Family highest education level was classified into less than high school, high school, and college or higher. Family income levels were classified according to the ratio of family income to federal poverty level (<1.0, 1.0-1.9, 2.0-3.9, and ≥4.0).

### Statistical Analysis

We used survey weights, strata, and primary sampling units created by the NCHS and provided along with the NHIS data in all the analyses, unless otherwise specified, so that the results are nationally representative of the US population. Briefly, survey weights used 4 weighting factors: inverse of the probability of selection, household nonresponse adjustment, first-stage ratio adjustment, and second-stage ratio adjustment.

Comparisons of characteristics among children with and without ASD were performed using a *t* test for continuous variables and a χ^2^ test for categorical variables. We estimated the odds ratios (ORs) and 95% confidence intervals of ASD according to the presence of allergic conditions using multivariable logistic regression, adjusting for age, sex, race/ethnicity, family highest education level, ratio of family income to federal poverty level, and geographic region. These factors are known to be associated with both allergic conditions and ASD. Because children with food allergy are more likely to have asthma and other allergies compared with children without food allergies,^26^ we performed a mutual adjustment for other allergic conditions.

To evaluate effect modification, we performed subgroup analyses according to age (3-11 or 12-17 years), sex (boys or girls), and race/ethnicity (white or nonwhite). Interactions between these factors and allergic conditions were tested by adding their multiplicative interaction terms in the multivariable models.

All data analyses were conducted using survey procedures of SAS statistical software version 9.4 (SAS Institute Inc). A 2-sided value of *P* < .05 was considered statistically significant.

## Results

Among the 199 520 children aged 3 to 17 years included in this analysis (unweighted mean [SD] age, 10.21 [4.41] years; 102 690 boys [51.47%]; 55 476 Hispanic [27.80%], 97 200 non-Hispanic white [48.72%], 30 760 non-Hispanic black [15.42%], and 16 084 non-Hispanic other race [8.06%]), 8734 had food allergy, 24 555 had respiratory allergy, and 19 399 had skin allergy. The weighted prevalence was 4.31% (95% CI, 4.20%-4.43%) for food allergy, 12.15% (95% CI, 11.92%-12.38%) for respiratory allergy, and 9.91% (95% CI, 9.72%-10.10%) for skin allergy. A diagnosis of ASD was reported in 1868 children (weighted prevalence, 0.95%; 95% CI, 0.89%-1.01%). Compared with children without ASD, children with ASD were more likely to be boys and to have higher family education levels (Table 1).

**Table 1.** Characteristics of 199 520 Participants According to Autism Spectrum Disorders (ASD) Status^a^

Variable	Children With ASD (n = 1868)	Children Without ASD (n = 197 652)
Age, mean (SD), y	9.78 (0.12)	10.01 (0.01)
Sex, No. (%)		
Boys	1478 (78.46)	101 212 (50.86)
Girls	390 (21.54)	96 440 (49.14)
Race/ethnicity, No. (%)		
Hispanic	386 (16.16)	55 090 (19.95)
Non-Hispanic white	1056 (62.23)	96 144 (58.19)
Non-Hispanic black	247 (12.85)	30 513 (14.36)
Other	179 (8.76)	15 905 (7.50)
Family highest education level, No. (%)		
Less than high school	217 (10.56)	43 462 (19.31)
High school	254 (13.92)	25 953 (13.02)
College or higher	1392 (75.33)	127 106 (67.14)
Missing	5 (0.19)	1131 (0.52)
Ratio of family income to federal poverty level, No. (%)		
<1.0	300 (16.01)	29 026 (14.65)
1.0-1.9	369 (20.40)	36 494 (18.16)
2.0-3.9	511 (27.35)	50 038 (25.80)
≥4.0	405 (20.55)	43 198 (22.14)
Missing	283 (15.69)	38 896 (19.25)
Geographic region, No. (%)		
Northeast	354 (18.76)	33 136 (17.39)
Midwest	412 (25.10)	40 332 (23.61)
South	602 (33.68)	71 792 (36.26)
West	500 (22.46)	52 392 (22.73)
Food allergy, No. (%)		
Yes	216 (11.25)	8518 (4.25)
No	1652 (88.75)	189 134 (95.75)
Respiratory allergy, No. (%)		
Yes	370 (18.73)	24 185 (12.08)
No	1498 (81.27)	173 467 (87.92)
Skin allergy, No. (%)		
Yes	312 (16.81)	19 087 (9.84)
No	1556 (83.19)	178 565 (90.16)

^a^ Data are presented as weighted means (standard deviations) for continuous variables, and frequencies (weighted percentages) for categorical variables.

Children with ASD, compared with those without ASD, were more likely to have food allergy (216 of 1868 [weighted prevalence, 11.25%] vs 8518 of 197 652 [weighted prevalence, 4.25%]; *P* < .001), respiratory allergy (370 of 1868 [weighted prevalence, 18.73%] vs 24 185 of 197 652 [weighted prevalence, 12.08%]; *P* < .001), and skin allergy (312 of 1868 [weighted prevalence, 16.81%] vs 19 087 of 197 652 [weighted prevalence, 9.84%]; *P* < .001). After adjustment for age, sex, race/ethnicity, family highest education level, family income level, and geographical region, the OR of ASD was more than doubled (OR, 2.72; 95% CI, 2.26-3.28; *P* < .001) among children with food allergy compared with those without food allergy. The magnitude of association with ASD was lower for respiratory allergy and skin allergy, but the associations were still statistically significant, with an OR of 1.53 (95% CI, 1.32-1.78; *P* < .001) for respiratory allergy and an OR of 1.80 (95% CI, 1.55-2.09; *P* < .001) for skin allergy. The observed associations were modestly attenuated but remained significant after mutual adjustment for other allergic conditions, with ORs of 2.29 (95% CI, 1.87-2.81) for food allergy, 1.28 (95% CI, 1.10-1.50) for respiratory allergy, and 1.50 (95% CI, 1.28-1.77) for skin allergy (Table 2). The adjusted OR of ASD was 1.82 (95% CI, 1.62-2.04; *P* < .001) among children with vs without any of these allergic conditions.

**Table 2.** Association of Food Allergy and Other Allergic Conditions With Autism Spectrum Disorders (ASD) in 199 520 Participants

Allergy Type	Children With ASD, No. (%) (n = 1868)^a^	Children Without ASD, No. (%) (n = 197 652)^a^	OR (95% CI)		
			Model 1^b^	Model 2^c^	Model 3^d^
Food allergy	216 (11.25)	8518 (4.25)	2.87 (2.39-3.45)	2.72 (2.26-3.28)	2.29 (1.87-2.81)
Respiratory allergy	370 (18.73)	24 185 (12.08)	1.59 (1.38-1.84)	1.53 (1.32-1.78)	1.28 (1.10-1.50)
Skin allergy	312 (16.81)	19 087 (9.84)	1.88 (1.62-2.19)	1.80 (1.55-2.09)	1.50 (1.28-1.77)

Abbreviation: OR, odds ratio.

^a^ Data are expressed as weighted prevalence of each allergic condition among children with or without ASD.

^b^ Model 1 adjusted for age and sex.

^c^ Model 2 adjusted for age, sex, race/ethnicity, family highest education level, ratio of family income to federal poverty level, and geographic region.

^d^ Model 3 adjusted for adjusted for age, sex, race/ethnicity, family highest education level, ratio of family income to federal poverty level, geographic region, and other allergic conditions.

In subgroup analyses, the association between food allergy and ASD was significant in all subgroups by age, sex, and race/ethnicity (Table 3). Respiratory allergy was not significantly associated with ASD in children aged 3 to 11 years, girls, or white children (Table 3). Skin allergy was not significantly associated with ASD in children aged 12 to 17 years or girls (Table 3). There was a significant interaction between race/ethnicity and respiratory allergy, with a stronger association in nonwhite children (OR, 1.79; 95% CI, 1.42-2.27) than white children (OR, 1.05; 95% CI, 0.85-1.31) (*P* = .004 for interaction). Sensitivity analyses including only children whose information was reported by their parents rather than other household members (n = 171 881) yielded similar results (eTable in the Supplement).

**Table 3.** Subgroup Analyses by Age, Sex, and Race/Ethnicity for Association of Food Allergy and Other Allergic Conditions With Autism Spectrum Disorders (ASD)

Variable	No. With ASD/Total Participants	Allergy Type								
		Food Allergy			Respiratory Allergy			Skin Allergy		
		OR (95% CI)^a^	*P* Value	*P* Value for Interaction	OR (95% CI)^a^	*P* Value	*P* Value for Interaction	OR (95% CI)^a^	*P* Value	*P* Value for Interaction
Age, y										
3-11	1136/114 535	2.20 (1.70-2.84)	<.001	.53	1.22 (0.99-1.50)	.07	.36	1.63 (1.32-2.01)	<.001	.44
12-17	732/84 985	2.52 (1.81-3.51)	<.001		1.36 (1.07-1.73)	.01		1.27 (0.97-1.66)	.08	
Sex										
Boys	1478/102 690	2.23 (1.78-2.79)	<.001	.67	1.26 (1.05-1.50)	.01	.79	1.55 (1.28-1.87)	<.001	.73
Girls	390/96 830	2.54 (1.66-3.89)	<.001		1.38 (0.99-1.92)	.06		1.37 (0.97-1.93)	.07	
Race/ethnicity										
White	1056/97 200	2.59 (2.01-3.33)	<.001	.45	1.05 (0.85-1.31)	.63	.004	1.42 (1.15-1.74)	.001	.23
Nonwhite	812/102 320	1.88 (1.33-2.65)	<.001		1.79 (1.42-2.27)	<.001		1.61 (1.23-2.10)	<.001	

Abbreviation: OR, odds ratio.

^a^ Multivariable model adjusted for age, sex, race/ethnicity, family highest education level, ratio of family income to federal poverty level, geographic region, and other allergic conditions.

## Discussion

In a nationally representative sample of US children, we found a significant and positive association of food allergy, respiratory allergy, and skin allergy with ASD. The association persisted after adjustment for demographic and socioeconomic variables and other types of allergic conditions. In addition, the association between food allergy and ASD was consistent and significant in all age, sex, and racial/ethnic subgroups.

Little is known about the association between food allergy and ASD. A previous case-control study among California children in the CHARGE (Childhood Autism Risks From Genetics and the Environment) study suggested that food allergy and sensitivities may be more common in children with ASD.^11^ Similar findings on food allergy and ASD were reported in another case-control study in northern California.^27^ Previous studies on allergic conditions and ASD have focused mainly on respiratory allergy and skin allergy. Several, but not all, of those studies reported a positive association of respiratory allergy (eg, asthma^15,28,29,30^) and skin allergy (eg, atopic dermatitis^14,31,32^) with ASD. The heterogeneity in previous studies may be partly due to limited sample size and statistical power.

In this large population-based study, all of these common allergic conditions were significantly associated with ASD. This indicates a possible presence of shared mechanisms (eg, immunologic dysfunction^8,9,10^) among these allergic conditions in relation to ASD. Indeed, immunological studies found that IgA, IgG, IgM, and total IgE were increased in children with ASD,^33^ and imbalance in T-cell subsets (low numbers of T_H_1-type interferon-γ and IL-2 positive cells) was also observed.^34^ Previous studies have shown increased levels of proinflammatory cytokines in the postmortem brains of patients with ASD, and increased autoantibody production has also been observed.^8,35,36,37,38,39^ It is possible that the immunologic disruptions may have processes beginning early in life, which then influence brain development and social functioning, leading to the development of ASD. In addition, there may also be shared genetic and nongenetic risk factors influencing both allergy and ASD.

Interestingly, the association between food allergy and ASD appeared stronger and more robust than the association of respiratory or skin allergy with ASD. The prevalence of both food allergy^13^ and ASD^2,3^ has increased over the past 2 decades. Although the underlying mechanisms for the observed association between food allergy and ASD remain to be elucidated, the gut-brain-behavior axis could be one of the potential mechanisms. Previous studies found higher prevalence of gastrointestinal symptoms among children with ASD.^40,41,42^ In addition, parents of autistic children reported more frequently their children having food allergies.^43,44^ Food allergy may involve alterations in the gut microbiome,^45^ allergic immune activation, and impaired brain function through neuroimmune interactions, which may finally affect the enteric nervous system and central nervous system leading to neurodevelopmental abnormalities.^16^ Of note, our results showed that the associations of respiratory allergy and skin allergy with ASD differed by population demographic characteristics, which may help explain the heterogeneous findings reported in previous studies.^14,15,28,29,30,31,32^

One major strength of this study is the use of nationwide population-based data with a large sample size and multiracial/multiethnic population. The nationally representative sampling strategy of the NHIS facilitates generalizing the findings to a broader population. In addition, the NHIS has a relatively high response rate,^25^ which reduces the concern of selection bias.

### Limitations

There are several limitations in this study. First, both allergic conditions and ASD were ascertained based on self-reported and retrospectively reported information, which may be subject to recall bias and misreporting. However, the prevalence of each allergic condition and ASD in this study was comparable to the numbers in other nationwide studies in similar years.^1,4,43^ In addition, clinical guidelines by the American Academy of Pediatrics recommend ASD-specific screening at age 18 to 24 months for all US children, which improves the identification of children with suspected ASD symptoms at a young age. Nonetheless, we could not rule out the possibility that some parents of children with ASD might overreport allergies in their children and some parents of children without ASD might underreport allergies in their children. Second, we did not know the onset timing of the allergic conditions or the timing of ASD diagnosis. Therefore, we cannot establish temporal relationship and causal inference from the current study. However, experimental evidence supports the notion that food allergy may increase the risk of ASD.^16,17^ Large prospective cohort studies starting from birth or early life are needed to confirm our findings. Third, the NHIS does not have laboratory data on specific IgE antibodies for the allergic conditions and therefore we were unable to determine the specific immunologic pathways for the observed associations.

## Conclusions

Based on nationally representative data in large cross-sectional surveys, we found a significant and positive association between common allergic conditions, especially food allergy, and ASD in US children. These findings warrant confirmation in prospective cohort studies. The underlying mechanisms for the association of food allergy and other allergic conditions with ASD remain to be elucidated.

## Notes

Supplement. **eTable.** Association of Food Allergy and Other Allergic Conditions With ASD, a Sensitivity Analysis by Restricting to Children (n = 171 881) Whose Information Was Reported by Their Parents Rather Than Other Household Members
