# Supplementary material for: Association of Food Allergy and Other Allergic Conditions With Autism Spectrum Disorder in Children
Source: JAMA Netw Open. 2018 Jun 8;1(2):e180279. doi: 10.1001/jamanetworkopen.2018.0279 (PMC6324407; doi:10.1001/jamanetworkopen.2018.0279)
Supplement: Supplement. — eTable. Association of Food Allergy and Other Allergic Conditions With ASD, a Sensitivity Analysis by Restricting to Children (n = 171 881) Whose Information Was Reported by Their Parents Rather Than Other Household Members [file jamanetwopen-1-e180279-s001.pdf]

Supplementary Online Content

Xu G, Snetselaar LG, Jing J, Liu B, Strathearn L, Bao W. Association of food allergy and other allergic conditions with autism spectrum disorder in children. *JAMA Netw Open*. 2018;1(2):e180279. doi:10.1001/jamanetworkopen.2018.0279

eTable. Association of Food Allergy and Other Allergic Conditions With ASD, a Sensitivity Analysis by Restricting to Children (n = 171 881) Whose Information Was Reported by Their Parents Rather Than Other Household Members

This supplementary material has been provided by the authors to give readers additional information about their work.

© 2018 Xu G et al. *JAMA Network Open.*

eTable. Association of food allergy and other allergic conditions with ASD, a sensitivity analysis by restricting to children (n= 171881) whose information was reported by their parents rather than other household members.

	Children without ASD	Children with ASD	Model 1[a]	Model 2[b]	Model 3[c]
Food allergy	4.35%[*]	11.53%[*]	2.87 (2.38-3.47)	2.73 (2.25-3.31)	2.32 (1.89-2.86)
Respiratory allergy	12.13%[*]	18.40%[*]	1.54 (1.33-1.80)	1.50 (1.28-1.75)	1.25 (1.06-1.47)
Skin allergy	10.11%[*]	17.01%[*]	1.85 (1.58-2.17)	1.77 (1.52-2.07)	1.49 (1.26-1.76)

ASD, autism spectrum disorders.

[*] Data are expressed as weighted prevalence of each allergic condition among children with or without ASD.

[a] Model 1: adjusted for age and sex.

[b] Model 2: model 1 plus race/ethnicity, family highest education level, family income to poverty ratio, and geographic region.

[b] Model 3: model 2 plus mutual adjustment for other allergic conditions as mentioned.

© 2018 Xu G et al. *JAMA Network Open.*
